# Mitochondrial Changes in Rat Brain Endothelial Cells Associated with Hepatic Encephalopathy: Relation to the Blood–Brain Barrier Dysfunction

**DOI:** 10.1007/s11064-022-03698-7

**Published:** 2022-08-02

**Authors:** Krzysztof Milewski, Karolina Orzeł-Gajowik, Magdalena Zielińska

**Affiliations:** Department of Neurotoxicology, Mossakowski Medical Research Institute, Polish Academy of Sciences, Pawińskiego St. 5, 02-106 Warsaw, Poland

**Keywords:** Hepatic encephalopathy, Endothelial cell, Blood–brain barrier, Mitochondria, Oxidative stress

## Abstract

The mechanisms underlying cerebral vascular dysfunction and edema during hepatic encephalopathy (HE) are unclear. Blood–brain barrier (BBB) impairment, resulting from increased vascular permeability, has been reported in acute and chronic HE. Mitochondrial dysfunction is a well-documented result of HE mainly affecting astrocytes, but much less so in the BBB-forming endothelial cells. Here we review literature reports and own experimental data obtained in HE models emphasizing alterations in mitochondrial dynamics and function as a possible contributor to the status of brain endothelial cell mitochondria in HE. Own studies on the expression of the mitochondrial fusion-fission controlling genes rendered HE animal model-dependent effects: increase of mitochondrial fusion controlling genes *opa1*, *mfn1* in cerebral vessels in ammonium acetate-induced hyperammonemia, but a decrease of the two former genes and increase of *fis1* in vessels in thioacetamide-induced HE. In endothelial cell line (RBE4) after 24 h ammonia and/or TNFα treatment, conditions mimicking crucial aspects of HE in vivo, we observed altered expression of mitochondrial fission/fusion genes: a decrease of *opa1*, *mfn1,* and, increase of the fission related *fis1* gene. The effect in vitro was paralleled by the generation of reactive oxygen species, decreased total antioxidant capacity, decreased mitochondrial membrane potential, as well as increased permeability of RBE4 cell monolayer to fluorescein isothiocyanate dextran. Electron microscopy documented enlarged mitochondria in the brain endothelial cells of rats in both in vivo models. Collectively, the here observed alterations of cerebral endothelial mitochondria are indicative of their fission, and decreased potential of endothelial mitochondria are likely to contribute to BBB dysfunction in HE.

## Hepatic Encephalopathy: A Brief Overview of Clinical Characteristics and Pathogenesis

Hepatic encephalopathy (HE) is a complex neuropsychiatric disorder that results from impaired liver function. As a consequence, insufficient clearance of toxins from blood, mainly ammonia, results in their accumulation in the brain. Impaired liver function, associated with acute, chronic liver failure or cirrhosis, often results in a wide range of neurological alterations, including cognitive and motor disturbances [1].

The cellular and molecular mechanisms underlying HE are complex and have not yet been fully deciphered. However, there is a consensus that ammonia, as a major neurotoxin, interferes with various aspects of brain metabolism and neural transmission, and impairs cerebral water-ion homeostasis [2, 3]. The above-listed abnormalities contribute to brain edema, the key pathologic manifestation of acute HE [4, 5]. The important role of inflammation in the pathogenesis of HE is being more and more emphasized [6–8].

In acute HE, brain edema leads to the patients’ death in more than 50% of cases, which is a consequence of increased intracranial pressure and herniation [4, 9]. While by analogy to other brain pathologies, brain edema associated with HE is likely elicited by a combination of cytotoxic and vasogenic factors their relative roles have long remained a matter of debate [4, 10].

The well-established view is that in brain astrocytes detoxify ammonia by an enzymatic reaction catalyzed by glutamine synthase [11]. The subsequent accumulation of glutamine most likely results in cellular edema formation, despite potential compensatory mechanisms. In agreement with the long-held view that HE is a primarily gliopathy [11, 12]. The development of brain edema in HE is believed to be primarily due to pathological cell swelling referred as cytotoxic edema. Cytotoxic component of brain edema in HE is thought to primarily reflect swelling of astrocytes by mechanisms related to intracellular metabolic and ion imbalance and the ensuing intracellular accumulation of water [13]. The molecular mechanisms underlying HE-induced cytotoxic edema have been relatively well delineated. Both clinical and animal model studies favor the direct role of ammonia in inducing cytotoxic components of brain edema. The current view proposes that ammonia induced astrocytic swelling by a complex interplay of (i) oxidative/nitrosative stress (ONS) (ii) impairment of import and export of osmotically active substances leading to intracellular osmotic imbalance, (iii) mitochondrial dysfunction related to excessive accumulation of ammonia-derived glutamine and successive intra-mitochondrial release of neurotoxic concentrations of ammonia [12, 14]. Some details warrant to be the highlight here.

In the astrocytic mitochondria, ammonia induces ONS by the formation of free radicals that in turn lead to the pathological condition called mitochondrial permeability transition pore (mPTP) [15]. The mPTP is characterized by a rapid loss of inner membrane potential and collapsed mitochondrial ATP-synthesis. Additionally, high intracellular Ca^2+^ concentration is considered to mediate mPTP and altered mitochondrial redox-state and pH [16]. Although mPTP has been extensively investigated due to its involvement in apoptosis in different diseases (e.g., traumatic brain injury, neurodegenerative diseases, and ischemia–reperfusion) the pore complex in the mitochondrial inner membrane responsible for the increase in permeability has not been structurally identified [17]. The occurrence of mPTP in ammonia-treated cultured astrocytes has been associated with cell volume increase suggesting its role in astrocyte swelling [18]. Furthermore, cultured astrocytes treated with ammonia and different cytokines: TNF-α, interleukin-1β, interleukin-6, and interferon-γ, presented the induction of the mPTP in a time-dependent and additive manner [19]. Studies on patients with HE have shown metabolic disturbances in the brain indicating a compromised oxidative metabolism most likely due to mitochondrial dysfunction, that were correlated with elevated levels of glutamine [20].

Experimental evidence underscores the contribution of the inflammatory component (peripheral or intracerebral) to cytotoxic brain edema [21, 22]. In turn, the term vasogenic brain edema reflects water accumulation in the extracellular space, and is related to its uncontrolled flux across a selective blood–brain barrier (BBB), in association with peripheral osmotically active substances. In contrast to cytotoxic edema, the role of the vasogenic component in the induction of brain edema in HE has been disputed [23]. The evidence is still contradictory and the underlying mechanisms remain obscure.

Below we discuss BBB alterations in HE pathology, and the discussion is preceded by a characterization of the composition and cytoarchitecture of BBB. Next, we discuss and present data regarding HE-related impairment in processes that contribute to endothelial dysfunction and culminate in increased BBB dysfunctionality, which is understood as a consequence of the disruption in mitochondrial quality control processes.

## Blood–Brain Barrier

The histological structure of the BBB is organized by the basement membrane, endothelial cells, pericytes, and astrocyte end-feet. The penetrability of this highly selective fastener between blood and CNS is controlled by two checkpoints: (1) the endothelial cells, whose cohesive properties and physical resistance of, supported by the inter-endothelial tight junction (TJ) proteins: zonula occludens-1 (ZO-1), claudin-5, occludin, junctional adhesion molecules (JAM); and, the adherents complexes, E-cadherin; and (2) the basal lamina. Both the endothelial cell permeability barrier and the basal lamina matrix derive from the cooperation between the endothelium and astrocytes and together constitute the BBB [24]. The endothelial cell barrier properties are only partly attributable to TJs proteins [25]; an important role is played by the stability of the endothelial cell–astrocyte assembly, which requires matrix adhesion. This view is supported by (i) high expression of integrins and dystroglycan at the BBB; (ii) correlation of alterations in the expression of integrins and dystroglycan with the degree of BBB permeability, and subsequent glial activation and neuronal injury; (iii) breakdown of the cerebral vasculature in transgenic mice in which specific integrins are absent [26]. The above considerations have collectively implicated that BBB consists of an array of different components, including TJs and the inter-endothelial adherens complexes (ACs), matrix adhesion complexes formed between adhesion receptors integrins on both endothelial cells and astrocytes, dystroglycan, a single heterodimeric transmembrane receptor, distinct from integrins that forms a physical link between the intracellular cytoskeleton and the extracellular matrix and anchors astrocytes (for reviews and references see del Zoppo and Milner [27]). Of note, vertical adhesion could be a central determinant of the endothelial portion of the intact BBB. This statement is supported by data indicating disturbed BBB permeability and decreased occludin-5 expression in mice after functional blocking of 1β-integrin [28].

Pericytes are important constituents of BBB regulating its functional aspects. While pericytes do not per se induce BBB-specific gene expression in endothelial cells, they inhibit the expression of molecules that increase vascular permeability and CNS immune cell infiltration [29]. Only recently, a link between pericytes’ loss and symptoms associated with neurologic diseases has begun to be elucidated [30, 31]. Uniquely positioned within the neurovascular unit between endothelial cells of brain capillaries, astrocytes, and neurons, pericytes regulate BBB formation and maintenance, vesicle trafficking in endothelial cells, vascular stability, capillary blood flow, and clearance of toxic cellular by-products necessary for normal functioning of the CNS [32]. Whether pericytes’ impairment contributes to BBB dysfunction in HE has not been addressed.

## Alteration of the BBB Function and Structure in HE

Fundamental ultrastructural analysis of the cerebral cortex of patients diagnosed with ALF reported by Kato et al., documented swelling of astrocytes end-feet and increased number of vacuoles and vesicles in endothelial cells and pericytes. The study also documented that the basement membrane were enlarged, with generalized rarefaction and vacuolization, while TJs of endothelial cells were intact [33]. The above findings were indicative of BBB disruption in ALF. Otherwise, the contribution of BBB impairment to HE has been a matter of contradictory reports. Rats with severe, acute HE induced by ip. injection of galactosamine and azoxymethane, have presented brain extravasation to Evans Blue and alpha-aminoisobutyric acid, the classical markers of BBB leakage and altered transport, respectively [34, 35]. The reduction in the expression of TJs proteins (occludin, claudin-5, ZO-1, -2) contributing to a reduction in the integrity of the BBB have become evident in brains from this rat models [36, 37]. On the other hand, in galactosamine-induced acute HE in the rabbit, capillary endothelial cells appeared normal, and no evidence of brain extravasation to horseradish peroxidase was observed [38]. Discrepant results have been obtained in animal models of acute HE based on hepatic devascularization [39, 40].

Reported inconsistencies in the observed BBB impairment may be due to differences in the use of animal species and/or acuteness vs chronicity of HE (etiology; toxins/surgical procedures). In a rat model of chronic HE induced by bile duct ligation (BDL), an electron microscopy study revealed anatomically intact BBB structure [41] and decomposition of ZO proteins expression [42]. Furthermore, in the same model, no brain extravasation of Evans blue or sodium fluorescein was found [9], nor any changes in the expression of the TJs proteins were detected. No breakdown of the BBB was likewise demonstrated in a rat model of chronic HE induced by portocaval anastomosis [43]. It is unknown, whether HE may lead to alterations in the matrix elements, matrix adhesion receptor expression by both endothelial cells and astrocytes, factors affecting vascular permeability. Concluding, alternations of the BBB resulting in permeability increase were reported in most of reproducible and well-characterized animal models of HE (Table 1).

**Table 1:** BBB related alternations observed in animal models of HE

Animal models of HE			Spec	BBB permeability	Findings	Genes or proteins level	Refs.
Acute	Toxin- induced models	Azoxymethane	Mice	↑		Claudin-5 ↓; MMP9 ↑ mRNA and protein level	[44–46]
		Acetaminophen	Mice	↑	Perivascular astrocytes swelling, extracellular space shrinkage	Occludin ↓protein level	[47]
		Carbon tetrachloride	Rat/mice	↑		ZO-1; VE-cadherin↓ protein level	[48]
		Galactosamine	Rat	Unaltered			[49]
			Rat	↑ In caudate-putamen, cerebellum, motor cortex, and globus pallidus, unchanged in frontal cortex or hippocampus	Vasogenic or cytotoxic edema		[34]
			Rat	Unaltered			[50]
			Rat	↑	Cerebral edema, perivascular astrocytes swelling		[51]
			Mice	↑	Tight junction disruption, extracellular space shrinkage, increased number of vesicles and vacuoles	Occludin ↓ protein level	[37]
			Rabbit	↑			[52]
		Thioacetamide	Mice	Unaltered			[53]
			Mice	↑			[45]
		Hepatic devascularization	Rat	↑	Vacuolization and swelling of perivascular astrocyte processes in cerebral cortex		[54]
	Surgical models	Hepatectomy	Rat	Unaltered			[55]
			Rat	↑	Amino acids active transport increase		[56]
Chronic	Toxin- induced models	Carbon tetrachloride	Mice	↑	Brain edema	Claudin-5; ZO-1; occludin↓ protein level MMP9 ↑ mRNA level	[57, 58]
	Surgical models	Portacaval anastomosis	Rat	↑	Plasma membrane folding of perivascular astrocytes, astrocytes swelling		[59]
			Rat	↑	Perivascular astrocytes swelling, increased number of vesicles in endothelial cells; HRP-devoid endothelial intercellular space		[60]
		Congenital portacaval shunts	Rat	Unaltered			[43]
			Dog	↑			[61]
		Graded portal vein stenosis	Rat	↑			[62]
		Bile duct ligation	Rat	↑	Microvessel integrity loss	Occludin, ZO-1, ZO-2 ↓ immunofluorescence	[63]

In the above mentioned studies chronic HE produced minimal HE and, therefore, it remains to be determined whether BBB breakdown is associated with overt HE. Interestingly, a magnetic resonance imaging study in cirrhotic patients demonstrated the co-existence of both cytotoxic and vasogenic brain edema [64]. Early works indicates a significant increase in the BBB permeability for ammonia [65], but subsequent studies have not substantiated alternations of the permeability-surface area of the BBB for ammonia in HE [66, 67]. Collectively, the role of increased permeability of the BBB in brain edema development and/or progression deserves more complex and detailed investigation (for discussion see Scott et al. [21]).

Importantly, some studies underscore the contribution of inflammatory factors to the vasogenic component of brain edema under HE. Inflammatory mediators in peripheral blood are not able to cross the BBB due to their molecular size. However, different types of cytokines can modulate endothelial TJs and thus unlock the BBB [68], or initiate endothelial inflammatory processes that involve downstream cyclooxygenase (COX), prostanoids, and NO signaling, allowing the direct interaction with astrocytes. Increased brain levels of TNFα, IL-1β, and IL6 and activation of microglia as a source for intracerebral cytokine production were reported in experimental models of ALF [69]. Upon systemic inflammation, microglial cells and astrocytes have been shown to release proinflammatory cytokines, which were suggested to contribute to enhanced neuropsychological impairment induced by hyperammonemia [11, 70, 71]. The study by Lv et al. suggested that deleterious effects of systemic inflammation for the brain are linked with the observed alterations in the BBB [37]. Mentioned study documented the crucial role of TNF-α in the development of BBB abnormalities and corroborated well with the observation of increased TNF-α in patients with ALF [37]. In acetaminophen-induced ALF mice, it was found that increases in BBB permeability positively correlated with elevated serum TNF-α levels, which could be prevented by administering anti-TNFα-IgG [47]. Similarly, TNF-α or TNF-α-R1 antibodies increased the expression of TJs proteins occludin and ZO-1, which result in decreased leakage of Evans Blue in brains of ALF mice induced by d-galactosamine and lipopolysaccharide [72]. Additionally, a possible mutual relation between neuroinflammation, cerebral blood flow, and intracranial hypertension have been suggested [73, 74].

Because cerebral endothelial dysfunction is often associated with compromised BBB, understanding the endothelial factors that regulate vessel function to maintain BBB role and prevent vascular permeability may provide insights into disease prevention and treatment.

## Mitochondrial-Derived Reactive Oxygen Species (ROS) in the Endothelial Cells: Implication to Cerebral Dysfunction Observed in HE

Endothelium relies predominantly on anaerobic glycolysis for ATP turnover and mitochondria make up 2–5% of the cytoplasmic volume of endothelial cells in most vascular units [75]. Besides its metabolic role mitochondria integrate signals from the environment, perceive cellular stresses, control cell death signaling to list a key function. Since mitochondria as a major source of oxidative stress contribute to the pathogenesis of HE, its role as a potential trigger of the brain endothelial dysfunction upon HE should be uncovered.

Mitochondrial ROS generation in the endothelial cells is considered as one of the primary cell signaling pathways. Endothelial cells produce different types of ROS, including superoxide (O_2_^−^), hydrogen peroxide (H_2_O_2_), peroxynitrite (ONOO^–^), hydroxyl radicals (OH^⋅^), and other reactive oxygen and nitrogen species [76, 77].

However, oxidative stress that results from increased oxidant production, reduced antioxidant capacity or both, leads to endothelial dysfunction by reducing nitric oxide (NO) bioavailability [78, 79]. Importantly, other sources of ROS in the vessels include NADPH oxidase (NOX) and xanthine oxidase [77].

Ammonia was found to cause the increased generation of ROS in rat brain endothelial cell line (RBE4) [80] and primary cultures of brain ECs [81]. In RBE4 cells, treatment with ammonia increased permeability of endothelial cells monolayer to fluorescein isothiocyanate (FITC)-dextran (40 kDa). This effect was ameliorated by co-treatment with a matrix metalloproteinase inhibitor, or an antioxidant, glutathione diethyl ester [80]. The matrix metalloproteinase 9 (MMP9) was also increased in the brain of BDL rats which accompanied an increase in permeability to sodium fluorescein, Evans blue, and FITC-dextran along with an increase in brain water content [82].

Increased levels of hydroxyl radicals were observed in rat brain vessels isolated from thioacetamide-induced HE [83]. An increase of hydroxyl radicals (OH^⋅^) was observed in rat brains in vivo after direct infusion of ammonium chloride to the striatum through a microdialysis probe [84]. Increased levels of nitrites and nitrates (markers of NO production) were also frequently detected in the brains of animals with experimentally induced HE [85–87]. Additionally, hyperammonemia in vivo was associated with increased expression and activity of heme oxygenase-1 (HO-1), a ubiquitous marker of oxidative stress [88, 89].

It has been shown that exogenously added l-glutamine reduces NO generation in the brain by inhibiting l-arginine transport via y + LAT2 exchanger. This effect was additionally potentiated after a direct infusion of ammonia to the brain via microdialysis probe [87], or when l-glutamine accumulated in there during HE [86]. Tentatively, the mechanism may also operate in the cerebral capillary endothelial cells forming the BBB, where enhanced glutamine level also would modulate l-arginine transport. Of note, increased expression of y + LAT2 was observed in RBE4 cells upon ammonia exposure [80], and in rat brains upon hyperammonemia in situ [86]. However, the outcome of carrier operation would depend on l-glutamine cell membrane gradient. The importance of this mechanism comes to light when confronted with the previous observation that l-glutamine infusion in the absence of hyperammonemia impairs cerebrovascular CO_2_ reactivity, most likely by reducing l-arginine availability and NO synthesis [90]. A recent study from our laboratory demonstrated strong differences in the reactivity of the middle cerebral arteries and in their response to extravascular l-arginine application between vessels isolated from rats with TAA-induced HE and control animals, implicating that impaired vascular tone of cerebral arteries, which may involve, among other factors, their persistent exposure to high l-glutamine [91].

Excess of ROS reduces NO bioavailability through mechanisms including NO scavenging that occurs when O_2_^−^ reacts with NO to form ONOO^−^, which broadly contributes to cellular ONS and uncoupling of endothelial nitric oxide synthase (eNOS) [92]. Moreover, dissociation of eNOS monomers in the uncoupled eNOS is associated with a higher ratio of eNOS monomer-to-eNOS dimers [83, 93]. In addition, O_2_^−^ can oxidize the essential eNOS cofactor BH4 to BH2, which subsequently leads to eNOS uncoupling whereby eNOS produces more O_2_^−^ and less NO and lead to inactivation of proteins through the nitration of tyrosine residues. Such proteins include the antioxidant enzyme MnSOD [94, 95].

In our very recent study, a decrease of eNOS content and its uncoupling concurred with and was likely causally related to, both increased brain content of ROS and decreased cerebral cortical blood flow (CBF) in the thioacetamide-induced animal model of acute HE [83].

The specific mechanisms by which mitochondria in the endothelium release ROS and uncouple eNOS involve mitochondrial ATP-dependent potassium channel (MitoK^+^ATP) activation and subsequent induction of mPTP. MitoK^+^ATP activity is regulated by falling ATP and rising ADP levels, thus linking cellular metabolism with membrane excitability [96].

It was recently reported, that the MitoK^+^ATP channel is involved in Parkinson’s disease (PD) mainly via the regulation of mitochondrial biogenesis and fission/fusion [97]. At the molecular level, the authors using in vivo and in vitro rotenone models of PD documented that the pore subunit of Kir6.1, the major component of the MitoK^+^ATP channel was the key contributor in its interaction with mitochondrial dynamics.

It is important to underline that mitochondria are both the source and a target of excess ROS. Excessive generation of ROS, particularly of ONOO^−^ can result in oxidative damage to the mitochondrial respiratory complexes [98]. Cytoplasmic ROS may elicit mitochondria depolarization, at least in part through the opening of MitoK^+^ATP channels, which results in mitochondrial ROS release by respiratory complexes and the mPTP. The release of mitochondrial ROS may further activate NADPH oxidase via protein kinase C, resulting in increased cytoplasmic O^−2^ production and reduced NO bioavailability [99]. On the other hand, hyperammonemia was also found to be associated with a decreased activity of antioxidant enzymes (glutathione peroxidase, superoxide dismutase, and catalase) in the brain, both in the cytosolic and mitochondrial fractions [100]. In line, glutathione accumulation in the extracellular space of rat prefrontal cortex upon ammonia infusion was previously documented [101]. Of note, manipulations resulting in the recovery of the enzyme activities of the GSH metabolism ameliorated HE symptoms both in experimental animals [102] and in human patients [103]. Moreover, excess ONOO^−^ can also lead to inactivation of the endogenous proteins, antioxidant mechanisms (i.e., mitochondrial SOD2) through nitration. Changes in nitrated proteins content supporting this scenario were observed in brain homogenates obtained from rats with TAA-induced acute HE [83]. The above data support the concept that ONS contribute to the alterations in BBB permeability and thus to the vasogenic component of cerebral edema associated with HE.

## Aberrant Mitochondrial Quality Control Linked to Endothelial Dysfunction

The role of mitochondria in brain endothelial cells has previously been underestimated since vascular endothelial cells are located in juxtaposition to types of cells, that heavily rely on oxidative phosphorylation. Such cells, which in the periphery are represented by skeletal muscle cells and cardiomyocytes, predominantly rely on anaerobic glycolysis for ATP turnover, and mitochondria make up 2–5% of their cytoplasmic volume [104].

The location of the mitochondria within the endothelial cell of different organs and locations differs, largely depending on the signaling required. For instance, in pulmonary artery endothelial cells where oxygen sensing is relevant, mitochondria are localized near the nucleus, ensuring hypoxia-induced transcriptional regulation [105]. In turn in coronary arterioles, endothelial mitochondria are anchored to the cytoskeleton, initiating vasodilation in response to shear stress [79].

**Fig. 1:**
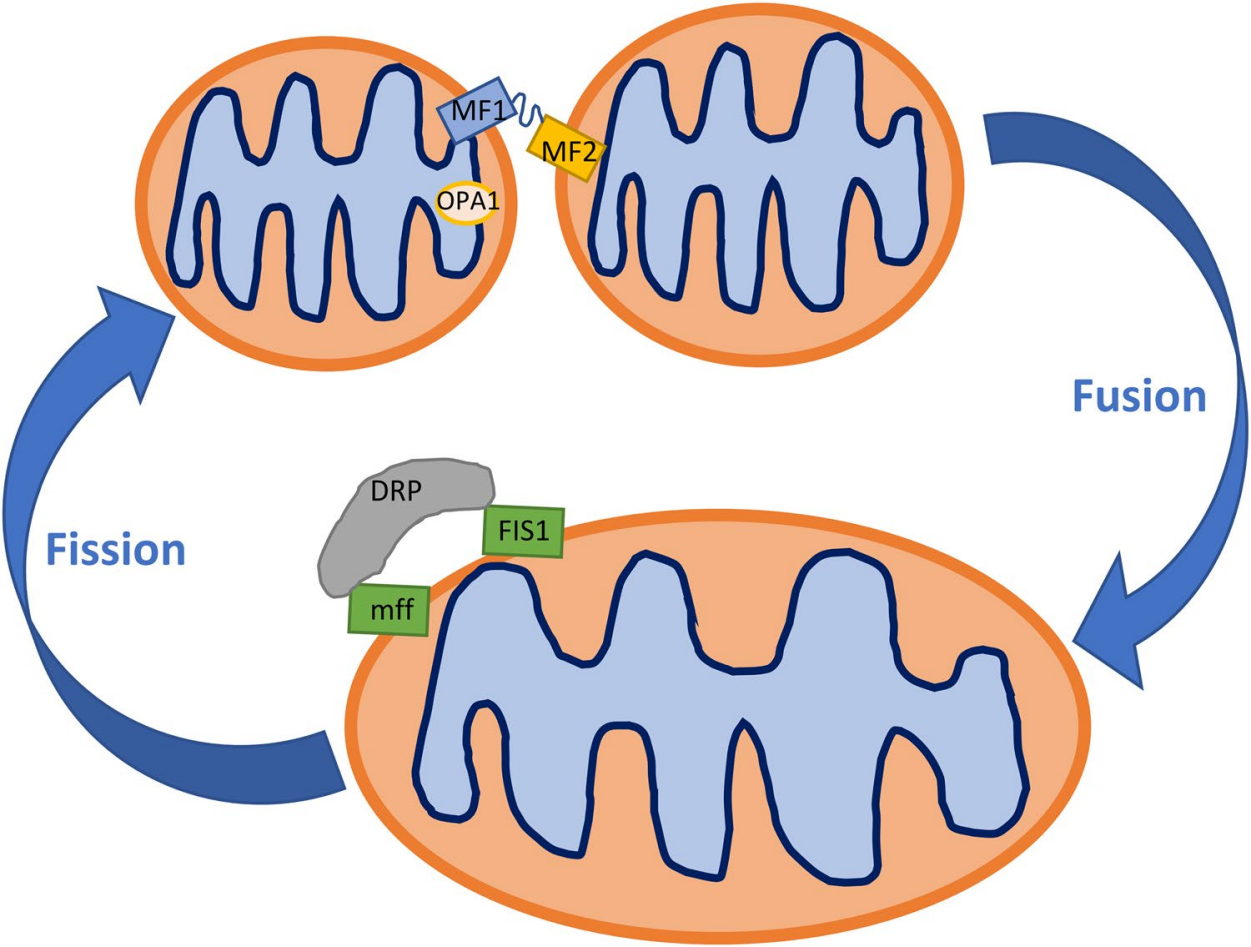
Schema of fusion/fission processes in the mitochondria. Mitofusin 1/2 and OPA1 are major proteins that control mitochondrial fusion. Mfn1 and Mfn2 locate in the outer mitochondrial membrane with their GTPase site facing the cytosol to coordinate the fusion process with the outer membrane of opposing mitochondria. OPA1 protein, localized in the intermembrane side, controls the fusion of the inner mitochondria membrane. DRP1, Mff, and Fis1 proteins regulated mitochondrial fission. DRP1 is localized in the cytosol and recruited to the outer mitochondrial membrane during fission. Fis1 and Mff are located in the outer mitochondrial membrane and work as the adaptor for DRP1

Cellular mitochondrial content is tightly regulated and is determined by the balance between mitochondrial biogenesis and degradation through the process called mitophagy, the form of autophagy in mitochondria (removal of damaged organelles). Mitochondrial organization, which is fundamental in determining their function, is determined by the balance between fusion and fission that determines the mitochondrial number, morphology, and size (Fig. 1) [106]. The cytoskeletal organization, which has a relevant role in maintaining mitochondrial network and function [107]. Mitochondrial quality control is required for optimal mitochondrial function, therefore dysregulation of these processes due to disease-related alterations initiate mitochondria-mediated cell senescence and apoptosis [78, 108]. In the brain, pathological state-associated disturbances in mitochondria quality control purportedly leading to cerebral endothelial dysfunction, and BBB impairment have gained attention only lastly [109]. In more general terms, the imbalance between controlling processes was documented and discussed in various disorders such as cancer, neurodegenerative and cardiovascular diseases [110–112].

Decreased mitochondrial biogenesis, one of the deregulatory events in the endothelium, is related to the regulator’s peroxisome proliferator-activated receptor-γ coactivator-1α (PGC-1α) and mitochondrial transcription factor A (TFAM) [113]. Further, dysregulation of mitochondrial dynamics is caused by an imbalance between proteins involved in fission [dynamin-related protein 1 (DRP1) and fission 1 (FIS1)] and fusion (transmembrane GTPases mitofusin 1 (MFN1) and 2 (MFN2) and the optic atrophy protein 1 (OPA1)). Mitochondrial fusion allows the transfer of gene products between mitochondria for proper functioning in normal conditions and specifically during metabolic and oxidative stress. In turn, mitochondrial fission is crucial for mitochondrial division and quality control. The role of mitochondrial failure was often described as associated with dysfunctional BBB in different neurologic disorders, including stroke, Alzheimer’s disease, Parkinson’s disease, Huntington’s disease, and epilepsy [114–119]. In so far, the role of altered mitochondrial dynamics involving fission, fusion, and mitophagy processes, has not been adequately explored in the context of BBB dysfunction upon HE.

## Effects of HE-Key Factors: Ammonia and TNFα on the Mitochondrial Membrane Potential, the Expression of Genes Involved in Mitochondrial Fusion and Fission, and Morphology of Mitochondria in Cerebral Endothelial Cell

**Fig. 2:**
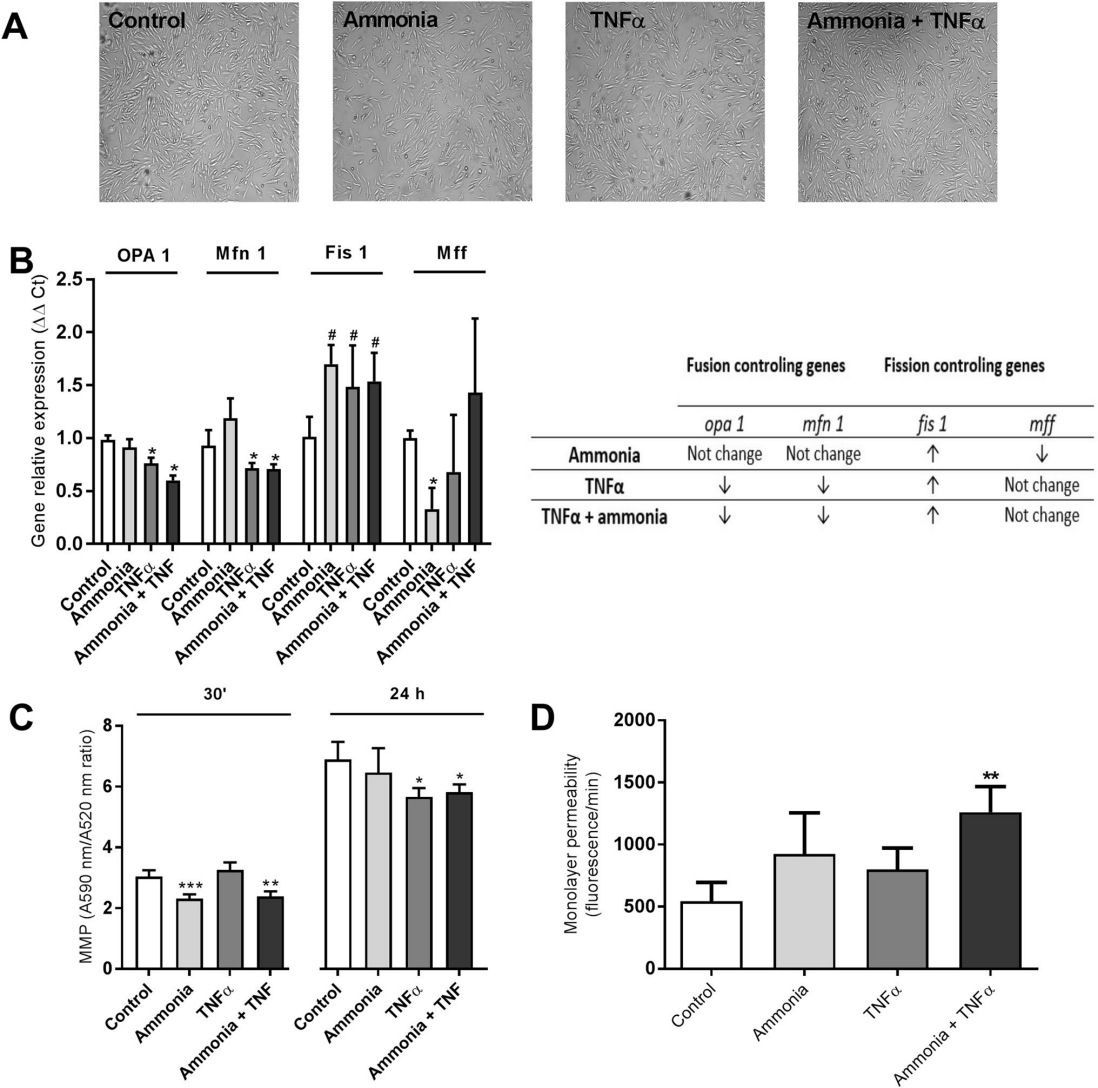
The effect of ammonia and/or TNFα treatment (5 mM ammonium chloride; 50 ng/ml TNFα; 24 h; Sigma-Aldrich; St. Louis, MO, USA) on RBE4 cell culture morphology and growth (**A**), mitochondrial gene expression (**B**), mitochondrial membrane potential (MMP) (**C**), monolayer permeability for fluorescein isothiocyanate (FITC) dextran 40 kDa (**D**). Relative gene expression was analyzed using the real-time PCR method followed by ΔΔCt quantification analysis in relation to beta actin gene expression. Probes for *opa1*(#Rn00592200_m1), *mf1* (#Rn00594496_m1), *fis1*(#Rn01480911_m1), *mff* (#Rn01400790_m1) and *actb* (Rn 0066789_m1) were purchased from Applied Biosystems, Waltham, USA. MMP was established using a Mitochondrial Membrane Potential kit, based on a JC-10 fluorescence probe, according to the manufacturer’s protocol (cat# MAK159, Sigma-Aldrich, Saint Louis, USA). Cells monolayer permeability for FITC-dextran (30 min exposition) was measured fluorometrically at 485/520 nm. Results are mean ± SD (n = 4); Two-way ANOVA test with Dunnett’s multiple comparisons test was performed using Graph Pad Software *p < 0.05 vs control; **p < 0.01 vs control; # 0.05 < p < 0.1 (tend toward significance) (panel **B**). One-way ANOVA test with Dunnett post-hoc test was performed using Graph Pad Software *p < 0.05 vs control; **p < 0.01 vs control; **p < 0.001 vs control (panel **C** and **D**)

We analyzed mitochondrial membrane potential and the expression of genes involved in mitochondrial fusion and fission in the rat brain endothelial cells (RBE4 cell line) treated with 5 mM ammonium chloride “ammonia” and 50 ng/ml of rat recombinant TNFα (Sigma-Aldrich, St, Louis, MO, USA). Both compounds were added into the cell culture medium for 24 h. We haven’t noticed any cell culture density changes or morphological alterations under inverted microscope (Fig. 2A). The expression analysis of genes coding mitochondrial fusion/fission proteins: *opa1*, *mfn1*, *fis1*, and *mff* (mitochondrial fission factor; MFF) revealed a decrease of *opa1*, *mfn1*, and, evident, but to be confirmed tendency to increase of the fission related *fis1* gene indicating disturbed fission process in the mitochondria of RBE4 cells upon treatments with both factors (Fig. 2B). Besides Fis1 participation in the mitochondrial fission via interactions with the Drp1, or by prevention of mitochondrial fusion through the inhibition of Mfn2/Opa1, Fis1 participates in mitophagy through recruitment of TBC1D15/17 and Syntaxin17 to mitochondria [120]. Fis1 is also proposed to interact with BAP31, inciting apoptosis [121]. Thus, Fis1 may act an important role in mitochondrial alternations during HE.

The measurement of mitochondrial membrane potential (MMP) revealed engrossing observation. MMP was decreased in both ammonia-treated groups after a short 30’ exposition. In turn, MMP was not significantly affected by ammonia but reduced in both TNFα- treated groups after 24 h (Fig. 2C). The short exposition of ammonia exerts probably toxic effects mainly by disturbing the pH balance. Ammonia in solution is present as NH_3_ or NH_4_^+^. NH_3_ is a weak base in gaseous form, since NH_4_^+^ is a weak acid. Because ammonia pKa is relatively high (approximately 9.2) most of total ammonia at physiologic pH is NH_4_^+^ [122, 123]. Ammonia toxicity results from disruption of the H^+^ gradient across the inner membranes of mitochondria. Due to relative alkalinity of the mitochondrial pH as compared to the cytoplasmic pH ammonia exits the mitochondrial matrix along this gradient and binds to H^+^ in the inter-membrane space, thereby eliminating the H^+^ gradient necessary for ATP synthesis [124]. In addition, NH_4_^+^ could compete with K^+^ ions at the K^+^ binding site of K^+^-channels and affect excitability and membrane potential in neurons [125]. Whether the same applies to endothelial mitochondria is not clear. In turn, the effects of TNFα may be linked with TNFα receptor activation and further signals transducing. In the endothelium, TNFα induces inflammatory responses by enhancing adhesion molecule expression and cytokine secretion [126, 127].

**Fig. 3:**
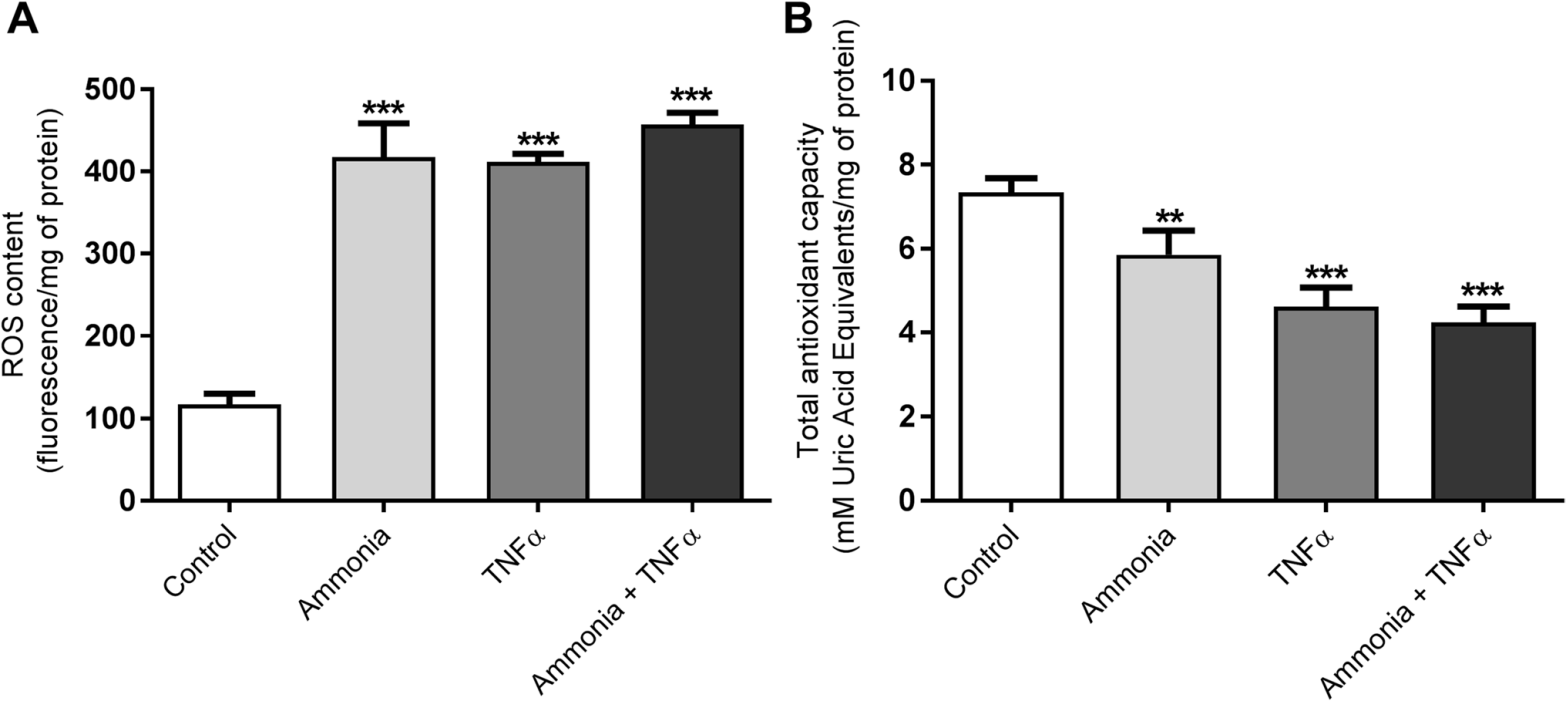
The effect of ammonia and/or TNFα treatment (5 mM ammonium chloride; 50 ng/ml TNFα; 24 h; Sigma-Aldrich; St. Louis, MO, USA) on the content of reactive oxygen species (ROS) (**A**) and total antioxidant capacity (TAC) (**B**). ROS levels were measured using the fluorescent probe 2′,7′-dichlorofluorescein diacetate (DCF-DA) 5 μM for 30 min at 37 °C. The fluorescence of cells was detected using Fluorescence Microplate Reader FLUOstar OMEGA (BMG Labtech, Ortenberg, Germany) with an excitation wavelength of 485/20 nm and emission wavelength of 528/20 nm. TAC was measured using the TAC Assay kit (cat# MAK187, Sigma-Aldrich, Saint Louise, USA) according to the manufacturer's instructions by the estimation of the capacity of the total antioxidants in the sample to convert Cu^2+^ in its reduced form, Cu^+^ which chelates with a colorimetric probe, giving a broad absorbance peak at ∼570 nm. Results are mean ± SD (n = 4); One-way ANOVA test with Dunnett post-hoc test was performed using Graph Pad Software **p < 0.01 vs control; ***p < 0.001 vs control

To verify if ammonia/TNFα treatment and observed mitochondrial impairment disturb endothelium function we measured cell monolayer permeability to isothiocyanate FITC-dextran. Simultaneous ammonia/TNFα administration caused a significant increase in permeability indicative of a disturbed barrier function of the endothelial cells (Fig. 2D). Experiments demonstrated an almost fourfold increase in ROS content in all treated cells with an accompanying decrease of total antioxidant capacity (TAC) especially, after simultaneous ammonia and TNFα treatment (Fig. 3).

We verified the morphology of mitochondria in cerebral vessels isolated from rats with hyperammonemia (OA) and thioacetamide (TAA)—induced acute liver failure. Both models were performed on male Sprague–Dawley rats (Tac: Cmd: SD, weight 200–220 g) supplied by the Animal House of Mossakowski Medical Research Centre, Warsaw, Poland (Approval no. 57/2015 (14 May 2015 from the 4th Local Ethics Committee for Animal Experimentation, Warsaw, Poland, as compliant with Polish Law). Briefly, hyperammonemia (OA) was induced by ip. injections of ammonium acetate (600 mg per kg) at 12 h intervals for three days. Acute liver failure was induced by thioacetamide (TAA) ip. administration (300 mg per kg at 24 intervals for three days). The control (sham) group received 0.3 mL of 0.9% NaCl (ip. for 3 days). Brain cortex microvessels were isolated on saccharose gradient as described earlier [63].

**Fig. 4:**
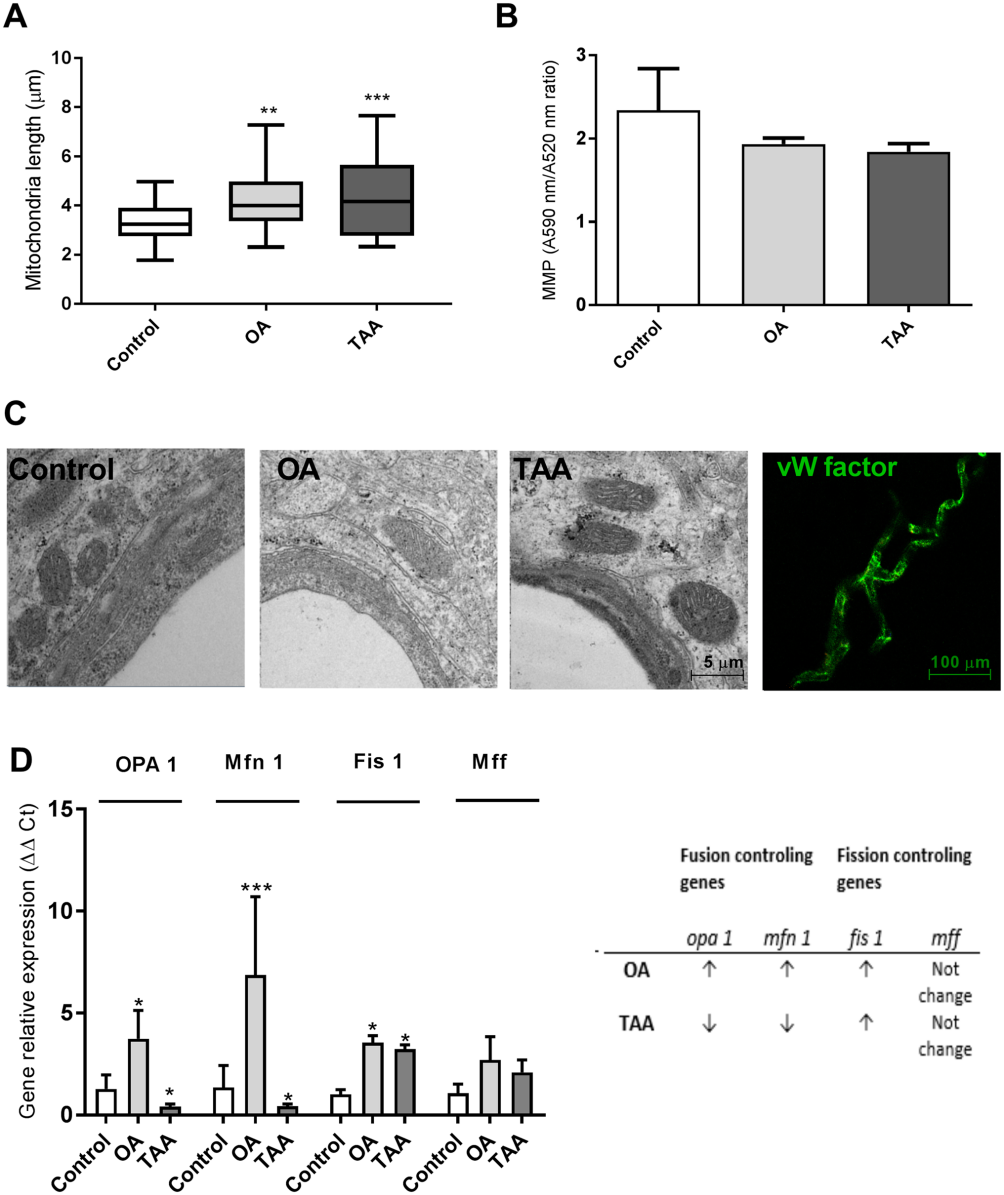
Mitochondria of cerebral vessels isolated from the brains of rats with hyperammonemia (OA) and thioacetamide (TAA)-induced acute liver failure. The length of the mitochondria in the brain vessels endothelium in the electron microscopy images was determined by quantitative evaluation using Digimizer Image Analysis Software (MedCalc Software Ltd, Ostend, Belgium) (**A**). Mitochondrial membrane potential (MMP) in cerebral vessels mitochondria of control, (OA), and (TAA)-administered rats was determined using Mitochondrial Membrane Potential kit (cat# MAK159, Sigma-Aldrich, Saint Louis, USA) (**B**). Representative electron microscopy images of cerebral vessel mitochondria and confocal microscopic image of vessels with von Willebrand factor immunostaining (**C**). Relative mitochondrial gene expression was measured using the real-time PCR method followed by ΔΔCt quantification analysis to beta-actin gene expression. Details of probes used are listed in the legend to Fig. 3 (**D**). All results are mean ± SD (n = 4); Two-way ANOVA test with Dunnett’s multiple comparisons test was performed using Graph Pad Software *p < 0.05 vs control; **p < 0.01 vs control (panel **B**). One-way ANOVA test with Dunnett post-hoc test was performed using Graph Pad Software *p < 0.05; **p < 0.01; ***p < 0.001 (panel **C** and **D**)

Electron microscopy documented enlarged mitochondria in the brain endothelial cells of OA and TAA rats suggesting mitochondrial swelling that occurs in both models (Fig. 4A), however, the mitochondria aspect ratio (width/length) evaluated using Digimizer Software (MedCalc Software Ltd., Ostend, Belgium) was unchanged. The mitochondrial membrane potential measurement in rat brain microvessels revealed a tendency toward a decrease in both experimental models which implies mitochondrial dysfunction leading to brain vessels energy depletion (Fig. 4B). The expression analysis of genes coding key fusion/fission proteins: *opa1*, *mfn*, *fis1,* and *mff* indicated possible increased fission of OA cerebral mitochondria and a decreased fusion of cerebral mitochondria from TAA rats, but further experiments are needed to prove this phenomenon (Fig. 4D), since gene expression changes are not necessarily reflected at the protein level.

## Roles of Mitochondrial Membrane Potential (Δψ) and Calcium for ROS Generation by the Mitochondria

The issues circumscribed by the title of this section have not been elaborated in our laboratory, but, definitely deserve comment. The role of mitochondrial membrane potential (Δψ) in ROS production is ambiguous. The complexity of the issue is underscored by findings variably associating ROS production with hyperpolarization and depolarization, likely depending on pharmacologically active agents and substrates being used, and the respiratory and cytoplasmic redox potential of the mitochondria. Selective MitoK^+^ATP openers that decrease Δψ in the cerebrovasculature do not increase ROS production [128].

The role of mitochondrial Δψ and ROS production in vascular dysfunction derives from data demonstrating that compared to healthy controls, arterioles and circulating mononuclear cells from patients with obesity and type 2 diabetes are characterized by mitochondrial membrane hyperpolarization (more negative Δψ), reduced mitochondrial mass, and greater ROS production [129–131]. Importantly, differences in mitochondrial membrane potential were observed as well. Mitochondrial alterations including membrane hyperpolarization reduced NO bioavailability and affected vascular function [103, 104]. In the former study, mitochondrial ROS production in monocytes was negatively correlated with artery flow-mediated dilation [129], a marker of endothelial function in humans.

Intracellular Ca^2+^ is essential for maintaining endothelium integrity and function [132]. In particular, the interaction of mitochondria with the endoplasmic reticulum is central in regulating intracellular Ca^2+^ levels, and this process directly involves mitochondrial Ca^2+^ uptake and cycling [133]. In addition to intracellular Ca^2+^ homeostasis control, mitochondrial Ca^2+^ levels play obvious roles in mitochondrial metabolism, cell signaling, biogenesis, and morphology [134], processes pertinent to optimal vascular function. In endothelial cells, increases in intracellular Ca^2+^ lead to eNOS activation and subsequent NO production [132]. As previously documented by our group, high levels of ammonia evoked a durable decrease of the basal intracellular Ca^2+^ level in RBE4 cells [135], which may contribute to cerebral vascular endothelial dysfunction associated with hyperammonemia and/or HE. The presence of ammonia-sensitive intracellular Ca^2+^ reservoirs has been previously described in bovine aortic endothelial cells [136]. However, the details of the evolution of Ca^2+^ to BBB dysfunction associated with HE have not been unraveled in those studies. Of note, increased Ca^2+^ efflux from brain mitochondria was observed in rats injected with ammonia [137].

Whether and how Ca^2+^ fluxes or other intermediary events occurring at the molecular or cellular level affect the function of cerebral blood vessels are translated into their functioning are intriguing questions worth further investigation.

## Conclusions

Investigations carried out to date revealed numerous potential links between various aspects of mitochondrial dynamics in rat brain endothelial cells and BBB dysfunction and its role in the pathogenesis of HE. However, the picture is far from being complete and the roads to the goal are filled with methodological obstacles. In terms of mitochondrial dynamics itself, the evidence regarding responses of its executors is still in a very preliminary stage. In terms of its link to BBB disruption, the status is obscured by a great model-to-model variability, which makes it difficult to translate the experimental results to clinical observations. Undoubtedly, an extension of the present knowledge on endothelial ammonia metabolism and how it affects the endothelial mitochondria is needed. Issues such as the role of glutamine metabolism and transport, operation of the glutamate/glutamine cycle, very well described in astrocytes, remain almost unaddressed concerning brain endothelial cells. Raising the level of knowledge of the above aspects of metabolism in HE-affected endothelial mitochondria to that already acquired about astrocytic mitochondria should help to unravel the secrets of BBB disruption and brain edema in HE.

## Data Availability

The data generated or analyzed during this study are available from the corresponding author upon reasonable request.

## Abbreviations

ALF: Acute liver failure
BBB: Blood–brain barrier
BDL: Bile duct ligation
BH4: Tetrahydrobiopterin
DCFH-DA: 2′,7′-Dichlorodihydrofluorescein diacetate
HE: Hepatic encephalopathy
MMP: Mitochondrial membrane potential
MMP9: Matrix metalloproteinase 9
mPTP: Mitochondrial permeability transition pore
NO: Nitric oxide
OA: Ammonium acetate/here used to refer to a rat model of hyperammonaemia
ROS: Reactive oxygen species
TAA: Thioacetamide
TAC: Total antioxidant capacity
TJs: Tight junctions
VE- Cadherin: Vascular endothelial-cadherin
ZO: Zonula occludens
