# Correction to: Mitochondrial Changes in Rat Brain Endothelial Cells Associated with Hepatic Encephalopathy: Relation to the Blood–Brain Barrier Dysfunction

**DOI:** 10.1007/s11064-022-03780-0

**Published:** 2022-10-08

**Authors:** Krzysztof Milewski, Karolina Orzeł‑Gajowik, Magdalena Zielińska

**Affiliations:** Department of Neurotoxicology, Mossakowski Medical Research Institute, Polish Academy of Sciences, Pawińskiego St. 5, 02-106 Warsaw, Poland

**Correction to: Neurochemical Research.**

10.1007/s11064-022-03698-7.

In the original version of this article, in funding information section, another funding information “Project is supported by ESF, POWR.03.02.00-00-I028/17 − 00” was missed to incorporate. The complete funding information should read as below:

Funding: This study was supported by the National Science Centre of the Republic of Poland (NCN) Grant number: 2015/19/B/NZ4/01902 and the statutory MMRI funds No.13. Additionally, the project is also supported by ESF, POWR.03.02.00-00-I028/17 − 00 as well.

